# Impact of *RANGAP1* SUMOylation on Smad4 nuclear export by bioinformatic analysis and cell assays

**DOI:** 10.17305/bb.2024.10443

**Published:** 2024-12-01

**Authors:** Feng Zhang, Jun Yang, Yifei Cheng

**Affiliations:** 1 Department of Pharmacy, Huashan Hospital, Fudan University, Shanghai, China; 2 Department of Anesthesiology, Huashan Hospital, Fudan University, Shanghai, China

**Keywords:** Glioma, *RANGAP1*, transforming growth factor (TGF)-β/Smad signaling pathway, SUMOylation, SUMO1

## Abstract

Small Ubiquitin-like Modifier (SUMOylation) regulates a variety of cellular activities, and its dysregulation has been associated with glioma etiology. The aim of this research was to clarify the function of SUMOylation-related genes in glioma and determine relevant prognostic markers. The Cancer Genome Atlas (TCGA) Glioma and GSE16011 datasets were analyzed through bioinformatics to identify Ran GTPase activating protein 1 (*RANGAP1*) as the hub gene for further study. Experimental validation consisted of quantitative real-time polymerase chain reaction (qRT-PCR), western blotting (WB), and immunoprecipitation (IP) to evaluate *RANGAP1* expression, function, and interaction with SUMO1. To assess the role of *RANGAP1* knockdown and SUMOylation in glioma cells, various assays were conducted, including cell proliferation, migration, invasion, and apoptosis. In addition, cell cycle analysis and immunofluorescence (IF) were performed. Through bioinformatics, *RANGAP1* was identified as a crucial prognostic gene for glioma. Experimental studies confirmed the downregulation of *RANGAP1* in glioma cells and verified that *RANGAP1* repair impedes tumor growth. When it comes to *RANGAP1* silencing, it enhanced cell proliferation, invasion, and migration. Additionally, SUMO1 was identified as a specific SUMO molecule coupled to *RANGAP1*, affecting the location of Sma and Mad-related protein 4 (Smad4) in the nucleocytoplasm and the transforming growth factor (TGF)-β/Smad signaling pathway. The functional impact of *RANGAP1* SUMOylation on cell proliferation and migration was further confirmed through experiments using a SUMOylation-impairing mutation (K524R). Our findings suggest that *RANGAP1* may be a potential prognostic marker in gliomas and could play a role in regulating cell proliferation, migration, and invasion. SUMOylation of *RANGAP1* is responsible for regulating the TGF-β/Smad signaling pathway, which is crucial for the progression of tumors. Further investigations and experiments are necessary to confirm these results.

## Introduction

Glioma is a common tumor arising in the brain and accounts for a large proportion of primary brain tumors [1]. These tumors can be classified based on their cell origin and grade, with glioblastoma multiforme (GBM) being the most aggressive type [2]. Environmental and genetic factors contribute to glioma pathogenesis, with exposure to ionizing radiation and certain genetic mutations identified as risk factors [3, 4]. The incidence and mortality rates of glioma vary globally, posing a considerable burden on public health [5]. Surgery, radiation, and chemotherapy are now available therapeutic methods; nonetheless, the outlook for gliomas remains poor due to their infiltrative nature and low treatment effectiveness [6]. Despite extensive research, challenges persist in understanding glioma biology, identifying reliable diagnostic markers, and developing effective treatments. The complex genetic and molecular landscape of gliomas underscores the necessity for continued investigation. Exploring novel diagnostic biomarkers, therapeutic interventions, and prognostic indicators is crucial to advancing glioma research and improving patient outcomes.

Post-translational modification known as SUMOylation, which is the covalent attachment of Small Ubiquitin-like Modifier (SUMO) proteins to particular target proteins, is a crucial process that regulates various cellular functions, such as subcellular localization, protein–protein interactions, and protein stability [7, 8]. The enzymatic cascade of SUMOylation includes activation by SUMO-activating enzymes (E1), conjugation by SUMO-conjugating enzymes (E2), and ligation by SUMO ligases (E3) [9]. The dynamic regulation, mediated by SUMO-specific proteases, underscores its intricate role in cellular regulatory networks, influencing functions like DNA repair, transcriptional regulation, and genome stability maintenance [10]. Dysregulation of SUMOylation has been implicated in various diseases, such as cancer and neurodegenerative disorders. In the context of glioma, Zhu et al. [11] revealed a distinctive relationship between SUMOylation and glioma, characterized by heightened prolyl cis/trans isomerase NIMA-interacting 1 (Pin1) expression leading to hyperSUMOylation of SUMO1-modified proteins in glioma stem cells. In another study, Zhang et al. [12] showed that downregulation of SUMO-specific protease 1 (SENP1) hindered glioma cell proliferation and migration while enhancing apoptosis. Additionally, Zhang et al. [13] highlighted the critical role of elevated SUMO1-modified protein SUMOylation, facilitated by prolyl-isomerase Pin1, in promoting glioblastoma malignancy. Furthermore, Yang et al. [14] identified the overexpression of SUMO-activating enzyme subunit 1 (SAE1) in glioma tissues, correlating with higher malignancy grades and poor overall survival (OS). SAE1 upregulation activated AKT SUMOylation-mediated signaling pathways, promoting glioma progression both in vitro and in vivo. The above examples demonstrate that an in-depth study of SUMOylation in the context of glioma can reveal its impact on disease progression and its potential therapeutic targets. This offers a crucial foundation for more research on the link between SUMOylation and essential genes in glioma.

In this study, we conducted bioinformatics analyses on the GSE16011 dataset and the Cancer Genome Atlas (TCGA)-glioma dataset using computational biology methods. Ran GTPase activating protein 1 (*RANGAP1*) was identified as a hub gene through these analyses. Subsequent in vitro experiments explored the interaction between *RANGAP1* and the Smad signaling pathway in brain glioma cells. Furthermore, we assessed the impact of *RANGAP1* and SUMO1 treatments on the growth of brain glioma cells and the Smad signaling pathway. These findings shed new light on the therapeutic implications for glioma treatment, providing valuable insights into regulating the effect of *RANGAP1* and SUMO1 throughout the course of glioma and their potential as targets for therapeutic interventions.

## Materials and methods

### Dataset download and processing

The GSE16011 dataset (https://www.ncbi.nlm.nih.gov/gds/?term=GSE16011), containing 277 tumor samples and their corresponding seven controls, was retrieved from the Gene Expression Omnibus (GEO) database (https://www.ncbi.nlm.nih.gov/gds/). Additionally, TCGA glioma dataset (https://tcga-data.nci.nih.gov/tcga/), with 666 tumor samples and five normal samples, was obtained from the ASSISTANT for Clinical Bioinformatic platform (https://www.aclbi.com/static/index.html). Differential gene expression analysis was conducted on both datasets using the Limma package in the R programming language (The R Foundation for Statistical Computing, Vienna, Austria). Under the criterion of *P* < 0.05, genes with a fold change (FC) greater than 1.5 were considered upregulated, while genes with an FC less than 0.67 were considered downregulated [15].

### Integration and functional enrichment analysis of intersection genes in glioma datasets

To identify intersectional differentially expressed genes (DEGs) in glioma, the intersection between up- or downregulated DEGs between the TCGA-glioma dataset and the GSE16011 dataset was analyzed using the Bioinformatics & Evolutionary Genomics website (http://bioinformatics.psb.ugent.be/webtools/Venn/). We downloaded 194 SUMOylation-related genes (Table S1) from the article “Novel risk model of three SUMOylation genes based on RNA expression for prediction of potential prognosis and treatment sensitivity of renal cancer.” The identified intersection genes were then further intersected with SUMOylation-related genes to derive a set of key intersection genes. Key intersection genes underwent enrichment analyses with the Kyoto Encyclopedia of Genes and Genomes (KEGG) and Gene Ontology (GO) using the Database for Annotation, Visualization, and Integrated Discovery (DAVID, https://david.ncifcrf.gov/) database; results with *P* < 0.05 were deemed statistically significant.

### Analysis of prognostic risk model of SUMOylation-related genes in glioma

The “glmnet” program in the R software was used to analyze overlapping genes. The tuning parameters for the Least Absolute Shrinkage and Selection Operator (LASSO) model within the “glmnet” package were determined through ten-fold cross-validation. The optimal *λ*, representing the minimum criterion for adjusted parameters, was identified to select the most predictive genes. These selected genes, representing the most statistically significant predictors in our dataset, formed the foundation of our prognostic model. Subsequently, the glioma cohort from the TCGA database was stratified into two risk groups based on the expression patterns of the relevant genes. A risk assessment was then conducted for both groups. Kaplan–Meier (KM) analysis was employed to determine the OS probability for the two risk groups. Additionally, the median survival time was calculated, and survival differences between the two groups were assessed using the log-rank test to derive *P* values. Hazard ratios (HRs) for the high-risk group were also computed to further elucidate relative risk. Ultimately, the “timeROC” software was used to create receiver operating characteristic (ROC) curves, and the area under curve (AUC) values were computed to assess how well the prognostic models predicted patient survival at one, three, and five years. Higher AUC values indicate stronger prognostic prediction capabilities.

### Screening of glioma hub genes

We used the KM plotter website (https://www.kmplot.com/) to perform survival analysis and determine the impact of 11 genes on OS probability in glioma patients. Log-rank *P* values were computed to quantify the statistical significance of observed differences. Subsequently, univariate and multivariate Cox regression analyses were then performed using the forest plot program to assess the prognostic value of the identified genes and clinical predictors (age, grade). The calculations included 95% confidence intervals (CIs), HRs, and *P* values for each variable. Variables with *P* values less than 0.05 were considered key prognostic factors. Following the identification of key prognostic factors, nomograms were constructed using the rms package to predict 1-, 3-, and 5-year survival rates. The C-index (concordance index) was calculated to assess the consistency of the predictions. Calibration curves were generated to visualize the ideal calibration of the nomogram, where closer alignment with the 1-, 3-, and 5-year survival curves indicated better predictive performance. Finally, the expression profiles of the three key genes were investigated using the GSE16011 dataset and TCGA glioma dataset. Wilcoxon tests were employed to assess variations in gene expression between TCGA glioma samples and normal control samples. Raw expression data were processed and visualized using the R programming language, specifically employing the ggplot2 package to generate boxplots illustrating the distribution of gene expression in tumor and normal samples.

### Cell lines and culture

The American Type Culture Collection (ATCC) provided the human brain glioma cells U251, SW1783, and U87 to our study. The cells were kept in Dulbecco’s Modified Eagle Medium (DMEM), which was enhanced with 10% fetal bovine serum (FBS) and 1% penicillin–streptomycin. HEK293T cells and Human normal brain glial cells (HEB) were also cultured under the same conditions. Cell cultures were maintained in a humidified environment with 5% CO_2_ at 37 ^∘^C.

### Transfection assay

U251 and U87 cells were seeded in 24-well plates at 2×10^5^ cells per well for transfection. Cells were transfected with three different siRNAs targeting *RANGAP1* (si-RANGAP1-1, si-RANGAP1-2, si-RANGAP1-3), with si-NC serving as the negative control. For the downregulation of the RANGAP1-SUMO1 complex, a specific siRNA (si-RANGAP1-SUMO1) was used. As directed by the manufacturer, Lipofectamine 3000 was used for the transfections. For experiments assessing the impact of SUMOylation inhibition, the SUMOylation inhibitors 2-D08 (150 µM for 24 h) and ML-792 (10 µM for 24 h) were applied to HEK293T cells.

### Plasmid transfection

The eukaryotic expression vector pTango-zeo was used to clone *RANGAP1* cDNA, creating the pFlag-RANGAP1 plasmid, which was validated by DNA sequencing. Co-transfection of pHA-UBC9, pFlag-RANGAP1, and His-tagged SUMO plasmids (pHis-SUMO1, pHis-SUMO2, pHis-SUMO3) was performed on HEK293T cells. U251 and U87 cells were treated with varying concentrations of ML-792 (0, 0.2, 0.5, and 1 µM) for 2 h to evaluate the dose-dependent effects on SUMOylation. Additionally, for 3 min, 100 ng/mL transforming growth factor (TGF)-β was used to activate U251 and U87 cells to investigate the activation of the TGF-β/Smad signaling pathway.

### Quantitative real-time polymerase chain reaction (qRT-PCR) assay

According to previous research methods, we conducted qRT-PCR experiments [16]. The following primer sequences were used in the amplification process: *RANGAP1* forward: 5′-GATCTCACTAGGGGAAGGACTC-3′, *RANGAP1* reverse: 5′-CACAGTTGTTGAGCTTGAGTTC-3′. SUMO1 forward: 5′-AAAGTCATTGGACAGGATAGCA-3′, SUMO1 reverse: 5′-TCTCTGACCCTCAAAGAGAAAC-3′. Similarly, the forward and reverse primers for *GAPDH* used as the reference gene, were as follows: *GAPDH* forward: 5′-ATTCCACCCATGGCAAATT-3′, *GAPDH* reverse: 5′-TGGGATTTCCATTGATGACAAG-3′.

### Western blotting (WB) assay

Protease and phosphatase inhibitors were included in RIPA lysis buffer (Thermo Fisher Scientific, USA), which was used to generate cell protein lysates. Cell fractions were prepared by following the manufacturer’s instructions for a nuclear/cytoplasmic extraction kit (Thermo Fisher Scientific, USA) in order to separate nuclear and cytoplasmic proteins. The BCA Protein Assay Kit (Thermo Fisher Scientific, USA) was utilized to ascertain the protein content. Proteins in equal quantities were separated using 10% SDS-PAGE and then put onto PVDF membranes from Millipore, USA. Membranes were blocked with 5% skim milk and incubated with primary antibodies (RANGAP1, CDK1, Cyclin B1, Cyclin A2, SUMO1, Smad2/3, Sma and Mad-related protein 4 (Smad4), HSP90, Lamin B1) (Abcam, 1:1000) and appropriate secondary antibodies. Among them, HSP90 and Lamin B1 were used as cytoplasmic and nuclear markers, respectively. As an internal reference, GAPDH (Abcam, 1:5000) was employed. Using a ChemiDoc imaging system (Bio-Rad, USA) and an enhanced chemiluminescence (ECL) kit (Thermo Fisher Scientific, USA), protein bands were seen and recorded.

### Immunoprecipitation (IP)

Following established protocols, cell lysates containing approximately 2 × 10^7^ cells were ready to extract all of the proteins from IP. To enrich the target protein, 50 µL of anti-Flag M2 affinity gel was incubated overnight at 4 ^∘^C with 1–2 mg of protein lysates. To capture the RANGAP1 protein, 2 mg of cellular supernatant was incubated overnight with anti-RANGAP1 antibody-bound protein-A beads. Immunoprecipitation using normal rabbit IgG was performed as a negative control to account for nonspecific protein binding. The protein complexes were extracted using the sample-loading buffer and then put through SDS-PAGE for WB detection after four washes with Tris-buffered saline (TBS) buffer.

### Immunofluorescence (IF) staining

For IF examination, U251 and U87 glioma cells were sown on glass coverslips in 24-well plates and left to adhere for 24 h. After being fixed for 15 min at room temperature in 4% paraformaldehyde, the cells were permeabilized for 10 min in phosphate-buffered saline (PBS) containing 0.1% Triton X-100. The cells were blocked for an hour with 5% BSA in PBS to prevent non-specific binding, and then they were incubated with primary antibodies against RANGAP1 and SUMO1 for an entire night at 4 ^∘^C. After incubating with primary antibodies, cells were treated three times with PBS before being incubated for 1 h at room temperature in the dark with the corresponding fluorescently tagged secondary antibodies. For 5 min, the nuclei underwent DAPI counterstaining. The coverslips were put onto glass slides using a fluorescent mounting medium following one more round of PBS washings. Confocal laser scanning microscopy was used to capture fluorescence images, and colocalization of RANGAP1 and SUMO1 was analyzed using the merge function of the imaging software by overlaying the respective signals.

### Cell counting kit-8 (CCK-8) assay

The proliferation of cells was assessed for vitality using the CCK-8 assay (Dojindo, Japan). In 96-well plates, U251 and U87 cells were planted at a density of 5 × 10^3^ cells per well. After adding the CCK-8 reagent to each well, a microplate reader (Thermo Fisher Scientific, USA) was used to measure the absorbance at 450 nm from 0 to 5 days of treatment.

### Cell invasion and migration assays

Transwell was used to assess cell invasion and migration. The upper chamber of the Transwell contained transfected brain glioma cells suspended in a serum-free medium. Following that, 10% FBS was added to the medium in the lower chamber of the Transwell. After an incubation period, cells with moving cell membranes were stained with DAPI and fixed with 4% paraformaldehyde. Lastly, inverted microscopy was applied to record the number of migratory cells in the field of view. The cell invasion studies were carried out as described previously, with matrigel covered in the upper chamber.

### Flow cytometry

For flow cytometric analysis, brain glioma cells were separated using trypsin-EDTA (Gibco, USA) and washed with PBS. To differentiate between live, apoptotic, and necrotic cells, they were stained with Annexin V and propidium iodide (PI) following the manufacturer’s instructions. For the analysis of the cell cycle, cells were fixed, treated with RNase, and stained with PI to evaluate the distribution across cell cycle phases. Analyses were conducted on a BD Biosciences flow cytometer, and the resulting data were analyzed with FlowJo software (FlowJo LLC, USA) to calculate the percentages of apoptotic cells and to ascertain the cell distribution during the various cell cycle phases.

### Ethical statement

This study utilized data from publicly accessible and de-identified databases, specifically the TCGA database (https://tcga-data.nci.nih.gov/tcga/) and the GEO database (https://www.ncbi.nlm.nih.gov/gds/?term=GSE16011). As these datasets are publicly available and do not contain any personal identifying information, no ethical approval was required for this study. This complies with the ethical guidelines and standards set forth by the relevant authorities and institutions.

### Statistical analysis

Statistical analysis was conducted using R software. Each experiment was replicated three times in our study, with quantitative data presented as mean ± standard deviation (SD). For normally distributed data, two-tailed Student’s *t*-tests were used to assess group differences; for non-parametric data, Wilcoxon tests were employed. Less than 0.05 was the threshold for statistical significance.

## Results

### Screening and enrichment analysis of SUMOylation-related genes in glioma

**Figure 1.**
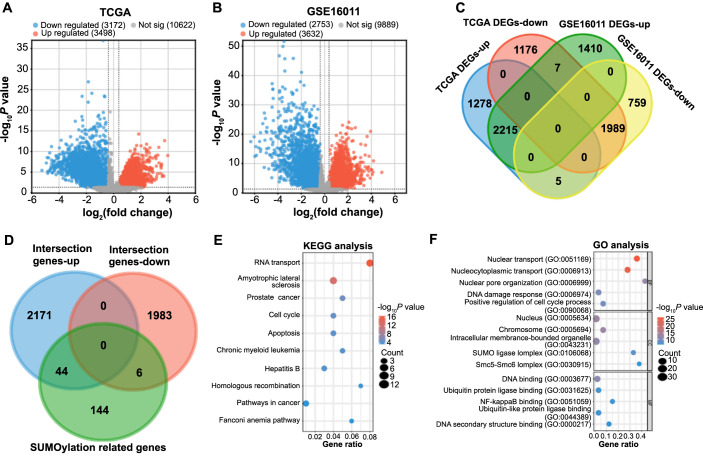
**Identification and enrichment analysis of SUMOylated genes in glioma.** (A and B) Volcano plots of DEGs for TCGA-glioma and GSE16011 datasets. Orange represents upregulated genes, blue represents downregulated genes, and gray represents insignificant genes; (C) Venn diagram obtained by the intersection of the upregulated DEGs of TCGA and the upregulated DEGs of the GSE16011 dataset, as well as the intersection of the downregulated DEGs of TCGA and the downregulated DEGs of the GSE16011 dataset; (D) Venn diagram obtained by the intersection of intersection genes-up, intersection genes-down, and SUMOylation-related genes; (E and F) Top 10 enriched KEGG pathways (E) and 15 GO terms (F). The *x*-axis represents the Gene Ratio; the *y*-axis represents the GO Term or enriched pathway; the size of the dots represents the odds ratio; the color of the dots represents the level of the *P* value. TGCA: The Cancer Genome Atlas; SUMO: Small Ubiquitin-like Modifier; DEGs: Differentially expressed genes; KEGG: Kyoto Encyclopedia of Genes and Genomes; GO: Gene Ontology.

Using the R package, we identified 3498 upregulated and 3172 downregulated DEGs from glioma samples and normal controls in the TCGA database. Similarly, the GSE16011 dataset yielded 3632 upregulated and 2753 downregulated DEGs (Figure 1A and 1B). Then, 2215 intersectional upregulated DEGs and 1989 intersectional downregulated DEGs were identified between the DEGs in TCGA and GSE16011 dataset (Figure 1C). Further intersection analysis identified 50 key intersection genes (Table S1) between these DEGs and 194 SUMOylation-related genes (Figure 1D). Subsequent functional enrichment analysis of 50 intersection genes using the DAVID database revealed significant enrichment of the Fanconi anemia pathway, cell cycle, Apoptosis pathways, etc. (Figure 1E). In GO term, key intersection genes were also related to SUMO Ligase Complex, DNA binding, NF-kappaB binding, and others (Figure 1F).

### Prognostic analysis of 11 signature genes

**Figure 2.**
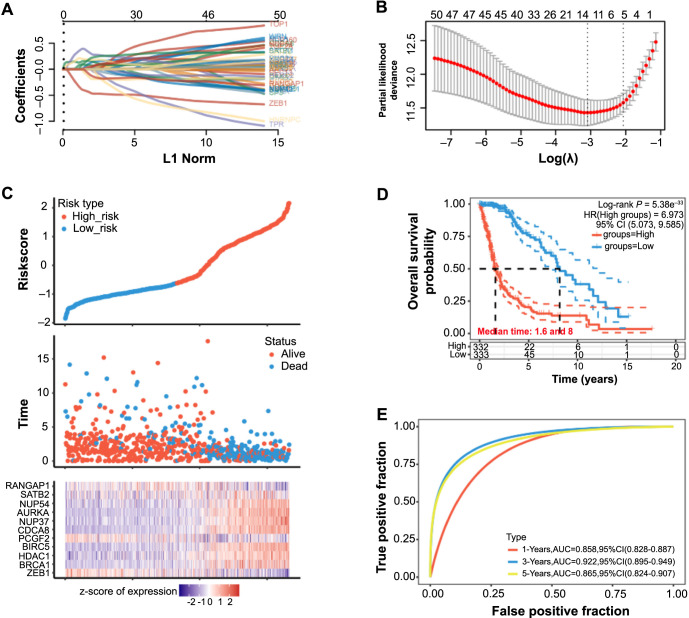
**Prognostic risk model analysis of SUMOylation-related genes in glioma.** (A) LASSO-Cox regression model analysis of SUMOylation-related genes in glioma, different colored lines represent different genes; (B) The relationship between 10-fold cross-validation partial likelihood deviation and log(λ). The leftmost vertical line represents the optimal lambda value that minimizes the cross-validation error, and the right vertical line represents the lambda value “within 1 standard error”; (C) Conduct risk model analysis on selected sample data. The upper image shows the risk score distribution of the high-risk group and the low-risk group, the middle image shows the survival status of different risk groups, and the lower image is a heat map of the cluster distribution of characteristic genes; (D) The KM survival curve analysis of the two groups in the risk model, the red line indicates the high-risk group and the green line indicates the low-risk group; (E) ROC curve analysis on the risk model in patients at 1, 3, and 5 years, the horizontal coordinate is a false-positive fraction, and the vertical coordinate is a true positive fraction. SUMO: Small Ubiquitin-like Modifier; LASSO: Least absolute shrinkage and selection operator; KM: Kaplan–Meier; ROC: Receiver operating characteristic; HR: Hazard ratio; CI: Confidence interval; AUC: Area under the curve.

Utilizing LASSO Cox regression analysis on the 50 intersection genes, we identified 11 signature prognostic genes with significant implications for glioma survival, determined by the optimal lambda value (lambda.min ═ 0.0454) (Figure 2A and 2B). The risk scores for these genes were computed as follows: Riskscore ═ (−0.4325)*ZEB1+(0.0999)*BRCA1+(0.0795)*HDAC1+(0.025)*BIRC5+(−0.1347)*PCGF2+(0.1656)*CDCA8+(0.3366)*NUP37+(0.1874)*AURKA+(0.0388)*NUP54+(0.1779)*SATB2+(−0.0785)*RANGAP1. In the risk model analysis, the high-risk group showed higher mortality and lower survival (Figure 2C). The results of KM survival analysis showed that the high-risk group had a median survival time of 1.6 years, while the low-risk group had a median survival time of eight years, and the high-risk group had a lower probability of OS (Figure 2D). ROC curve analysis further underscored the predictive accuracy of the risk model, with maximum effectiveness observed at three years (AUC ═ 0.922).

### Identification of prognostic genes in glioma and selection of *RANGAP1* as a potential therapeutic target

**Figure 3.**
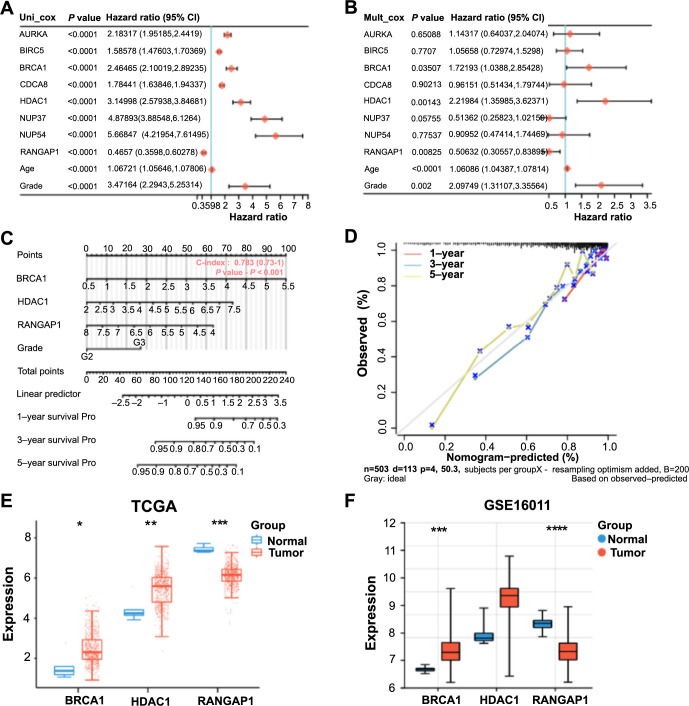
**Prognostic analysis of gene expression and clinical factors in glioma.** (A and B) Univariate/multifactorial Cox regression analysis on signature genes and clinical prognostic variables. The significance of each factor in relation to overall survival is indicated by the *P* value; (C) Nomogram of key prognostic variables and C-index used to evaluate the predictive ability of the model at 1-, 3-, and 5-years survival; (D) The dashed line indicates the ideal calibration curve of the nomogram with the red dash, blue dash, and yellow dash for the 1-year, 3-year, and 5-year predictions, respectively; (E and F) Box plot of expression analysis of three key genes in TCGA-glioma and GSE16011 dataset. Among them, blue represents normal samples and red represents tumor samples. *P < 0.05, **P < 0.01, ***P < 0.001, ****P < 0.0001. TGCA: The Cancer Genome Atlas; Mult_cox: Multivariate Cox regression analysis; Uni_cox: Univariate Cox regression analysis.

**Figure S1.**
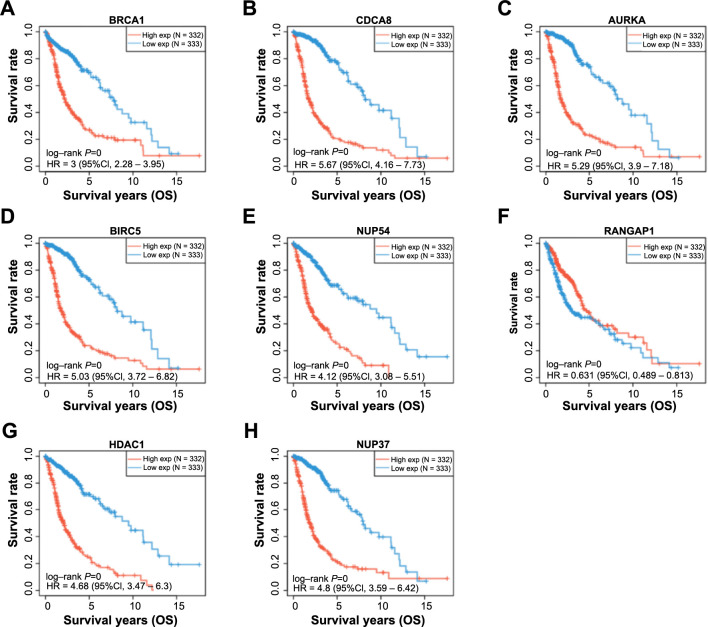
**OS prognosis of 8 prognostically significant genes in glioma.** (A–H) KM survival curve chart, OS probability of patients with high and low gene expression during 15 years. Red represents high expression and blue represents low expression. Genes analyzed were as follows: *BRCA1* (A), *CDCAB8* (B), *AURKA* (C), *BIRC5* (D), *NUP54* (E), *RANGAP1* (F), *HDAC1* (G), and *NUP37* (H). KM: Kaplan–Meier; OS: Overall survival.

In the OS prognosis analysis of the 11 selected signature genes, eight genes exhibited significant prognostic implications (Figure S1). Analysis of eight genes and two clinical variables in the risk model identified three statistically significant genes (*P* < 0.05): *BRCA1*, *HDAC1*, and *RANGAP1* (Figure 3A and 3B). Nomogram analysis demonstrated that these variables possessed significant predictive power for patients’ 1-, 3-, and 5-year survival rates, supported by the calibration curve results (Figure 3C and 3D). How these three genes in TCGA-glioma and GSE16011 datasets are depicted in Figure 3E and 3F. *BRCA1* and *HDAC1* exhibited significantly elevated expression in tumor samples, while *RANGAP1* demonstrated significantly decreased expression in tumor samples. Therefore, due to its prognostic significance and unique expression characteristics, *RANGAP1* was selected as the hub gene for subsequent analysis.

### Downregulation of *RANGAP1* promotes glioma cell growth

**Figure 4.**
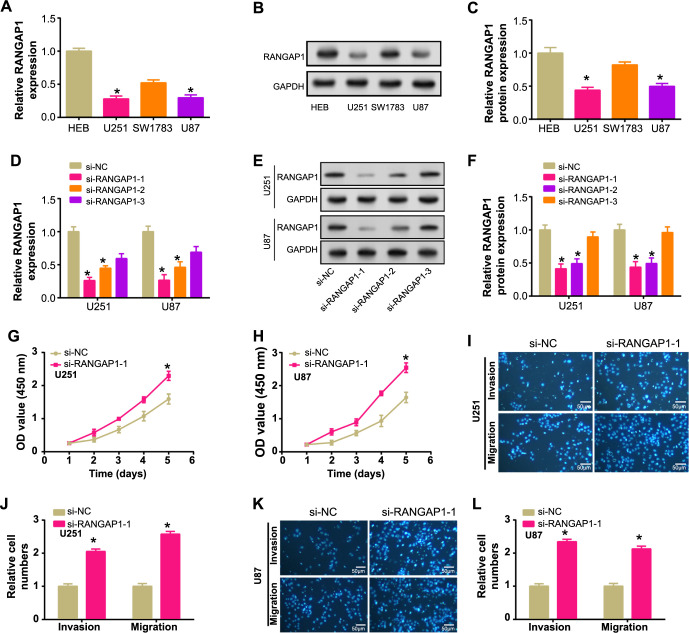
**Downregulation of RANGAP1 promotes proliferation, migration, and invasion of glioma cells.** (A–C) qRT-PCR and WB detected the expression of RANGAP1 in (HEB) and glioma cells (U251, SW1783, and U87); (D–F) qRT-PCR and WB detected the knockdown efficiency of RANGAP1(si-RANGAP1-1, si-RANGAP1-2, si-RANGAP1-3) in glioma cells (U251 and U87); (G and H) CCK8 detects the effect of si-RANGAP1-1 on U251 and U87 cell proliferation. Green represents si-NC group, and red represents si-RANGAP-1 group; (I–L) Transwell detects the effect of si-RANGAP1-1 on the invasion and migration numbers of U251 and U87 cells. Scale bar is 50 µm. *P < 0.05. Each experiment was replicated three times. qRT-PCR: Quantitative real-time polymerase chain reaction; WB: Western blotting; CCK-8: Cell counting kit-8; HEB: Normal brain glial cells; NC: Normal control group.

qRT-PCR revealed a significant downregulation of *RANGAP1* in glioma cells, which was further corroborated by WB analysis indicating lower protein levels (Figure 4A–4C). Subsequently, *RANGAP1* knockdown experiments were conducted in U251 and U87 cells, evaluating knockdown efficiency. si-RANGAP1-1 demonstrated superior knockdown efficiency, and it was selected for subsequent experiments (Figure 4D–4F). Functional assays, such as CCK-8 viability and Transwell assays, revealed that the induction of si-RANGAP1-1 significantly enhanced cell proliferation, viability, invasion, and migration abilities (Figure 4G–4L).

### *RANGAP1* knockdown disrupts cell cycle distribution and inhibits apoptosis in glioma cells

**Figure 5.**
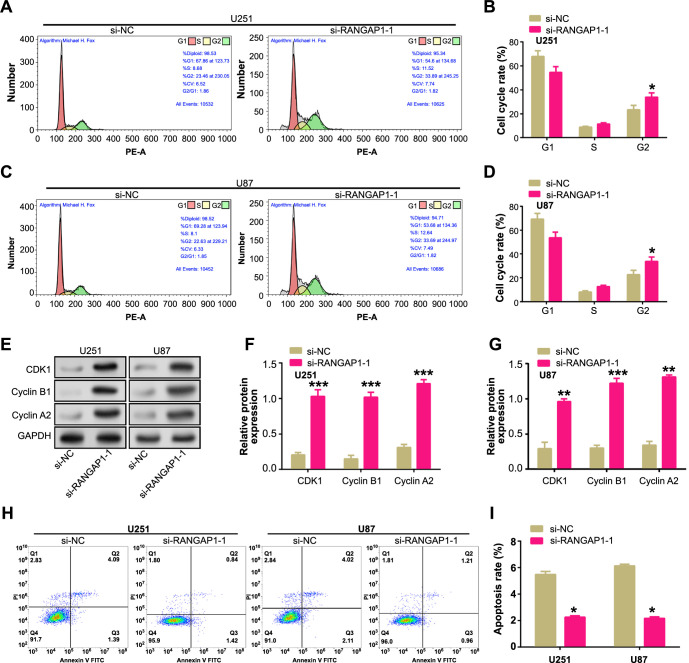
**RANGAP1 knockdown disrupts cell cycle distribution and inhibits apoptosis in U251 and U87 cells**. (A–D) Flow cytometry to detect the effect of si-RANGAP1-1 on the cell cycle of U251 and U87 cells; (E–G) WB detection of the effect of si-RANGAP1-1 on cell cycle proteins (CDK1, Cyclin B1, Cyclin A2) in U251 and U87 cells; (H and I) Flow cytometry to detect the effect of si-RANGAP1-1 on apoptosis of U251 and U87 cells. *P < 0.05. Each experiment was replicated three times. WB: Western blotting.

The functional role of *RANGAP1* in cell cycle distribution and apoptosis was investigated by downregulating *RANGAP1* in glioma cells and analyzing the effects using flow cytometry. The results revealed a significant accumulation of cells in the G2 phase of the cell cycle upon knockdown of *RANGAP1* (Figure 5A–5D). Western blot analysis further demonstrated that si-RANGAP1-1 led to a marked increase in the expression levels of cell cycle-related proteins, including CDK1, Cyclin B1, and Cyclin A2, in both U251 and U87 cells (Figure 5E–5G). Additionally, flow cytometry analysis indicated a corresponding decrease in the rate of apoptosis following *RANGAP1* knockdown in U251 and U87 cells (Figure 5H and 5I). These findings collectively suggested that *RANGAP1* played a crucial role in regulating the cell cycle and programmed cell death in glioma cells.

### SUMO1 conjugation modifies *RANGAP1*

**Figure 6.**
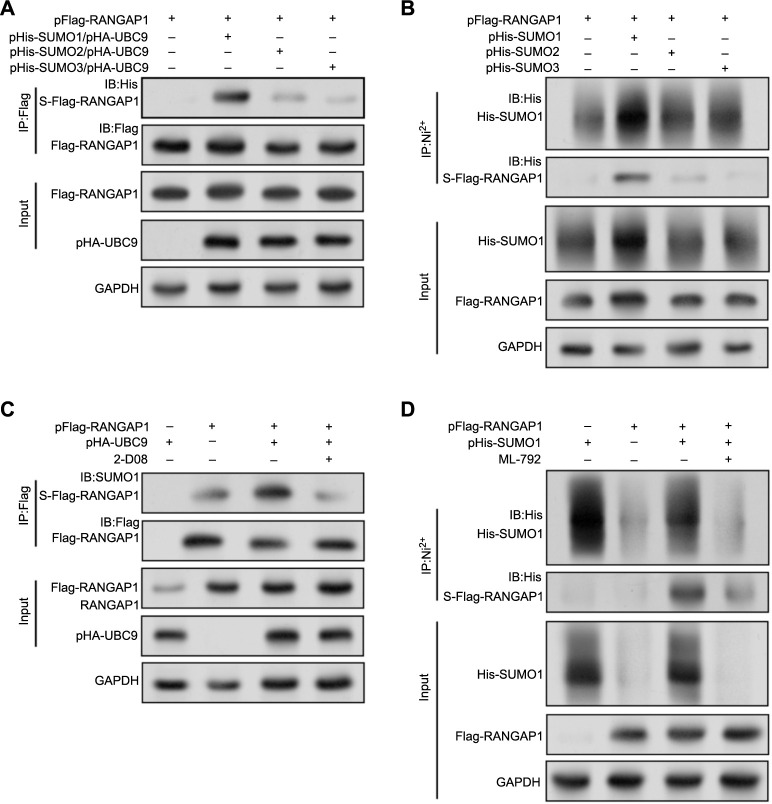
**SUMO1 conjugation modifies RANGAP1.** (A) WB analysis of RANGAP1 SUMOylation following co-immunoprecipitation with HA-tagged UBC9 and His-tagged SUMO proteins in HEK293T cells. Anti-His antibody detects SUMOylated RANGAP1 after Flag immunoprecipitation. The lower panels show total RANGAP1 and HA-UBC9 levels in the initial lysates with GAPDH serving as a loading control; (B) His-tag affinity purification of SUMOylated proteins demonstrates the specific SUMOylation of Flag-RANGAP1 in the presence of His-SUMO1, His-SUMO2, and His-SUMO3; (C and D) The SUMOylation of RANGAP1 was examined in response to UBC9/SUMO1 enhancement or inhibitor treatment (2-D08 or ML-792). HEK293T cells were co-transfected with pFlag-RANGAP1 and pHA-UBC9 or pHis-SUMO1 plasmids, followed by treatment with 150 µM 2-D08 (C) or 10-µM ML-792 (D) for an additional 24 h. Immunoprecipitation using anti-Flag antibody-coupled agarose beads (C) or Ni2±NTA agarose beads (D) enriched the Flag-tagged RANGAP1 protein, allowing analysis of SUMOylated RANGAP1 levels by WB assay using Flag or SUMO1 antibodies. S-Flag-RANGAP1 denotes SUMOylated Flag-tagged RANGAP1, and Input refers to an equivalent amount of cell lysate loaded. 2-D08 or ML-792 serves as a SUMOylation inhibitor. Each experiment was replicated three times. WB: Western blotting; SUMO: Small ubiquitin-like modifier; Flag: A short peptide sequence.

To identify the specific type of SUMO molecule that conjugates with *RANGAP1*, three exogenous His-tagged SUMO plasmids, and the pFlag-RANGAP1 and pHA-UBC9 plasmids were co-transfected into HEK293T cells. Immunoprecipitation with anti-flag antibody beads revealed that *RANGAP1* preferentially forms a complex with UBC9 in the presence of SUMO1. The interaction of SUMO2 and SUMO3 was significantly reduced, indicating a higher specificity of SUMO1 modification (Figure 6A). Similarly, Ni2±NTA agarose bead purification of His-tagged SUMO conjugates confirmed significant SUMOylation of *RANGAP1* in cells co-transfected with SUMO1, as opposed to SUMO2 or SUMO3 (Figure 6B). Further investigations into the impact of chemical SUMOylation inhibitors on *RANGAP1* SUMOylation levels were conducted. Transient transfection of pFlag-RANGAP1 plasmids in HEK293T cells resulted in obvious SUMOylation of exogenously expressed *RANGAP1* (Figure 6C). Upon UBC9 stimulation, the abundance of SUMOylated *RANGAP1* significantly increased. However, treatment with 150 µM inhibitor 2-D08 for 24 h led to a significant reduction in SUMOylated RANGAP1, despite the presence of UBC9, attributed to 2-D08 interference with the binding of UBC9 with SUMO1. Additionally, *RANGAP1* SUMOylation was also abolished with another SUMOylation inhibitor, ML-792 (10 µM, 24 h) treatment (Figure 6D). Taken together, these results collectively demonstrate that *RANGAP1* was modified through SUMO1 conjugation, which modification is dynamically regulated by SUMO-specific proteases and can be inhibited by 2-D08 or ML-792, emphasizing the specificity and reversibility of *RANGAP1* SUMOylation.

**Figure 7.**
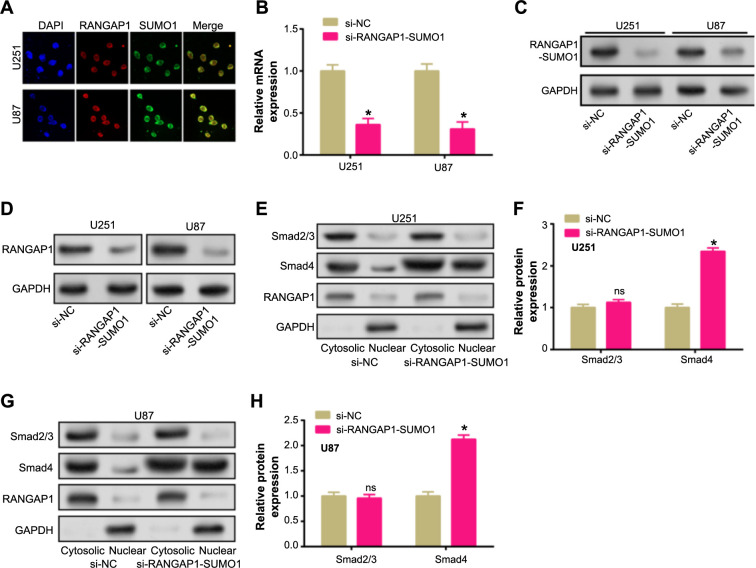
**Knockdown of SUMO1 affects the nucleocytoplasmic distribution of Smad4 in the TGF-β/Smad signaling pathway.** (A) Immunofluorescence staining of U251 and U87 cells displaying the localization of RANGAP1 (red), SUMO1 (green), and the nucleus (DAPI, blue). The merged images show the colocalization of RANGAP1 and SUMO1 within the cells; (B and C) qRT-PCR and WB detected the transfection efficiency of siRNA targeting RANGAP1-SUMO1 complex (si-RANGAP1-SUMO1) in glioma cells; (D) WB detection of RANGAP1 expression changes after RANGAP1-SUMO1 complex knockdown in glioma cells (U251 and U87); (E–H) WB detects the expression changes of Smad2/3 and Smad4 in the nucleoplasm of U251 and U87 cells after RANGAP1-SUMO1 complex knockdown. ns: not significant, *P < 0.05. Each experiment was replicated three times. qRT-PCR: Quantitative real-time polymerase chain reaction; WB: Western blotting; Smad4: Sma and Mad-related protein 4; TGF: Transforming growth factor; SUMO: Small Ubiquitin-like Modifier; IF: Immunofluorescence; ns: Not significant; NC: Normal control group.

Within the TGF-β/Smad signaling cascade, the RANGAP1-SUMO1 complex influences the nucleocytoplasmic distribution of Smad4. IF staining results showed a significant correlation between SUMO and RANGAP1 in U87 and U251 cells. Confocal microscopy images further revealed partial colocalization of SUMO1 and RANGAP1, mainly within the nuclei of glioma cells (Figure 7A). *RANGAP1*-SUMO1 complex knockdown effectiveness was confirmed by WB analysis and qRT-PCR (Figure 7B and 7C). With the knockdown of the *RANGAP1*-SUMO1 complex, the protein level of RANGAP1 was also significantly reduced (Figure 7D). The effect of RANGAP1-SUMO1 knockdown on Smad2/3 and Smad4 localization was subsequently analyzed. In U251 cells, we observed that Smad2/3 was mainly retained in the cytoplasmic fraction, whereas Smad4 showed a significant increase in the nuclear fraction after knockdown (Figure 7E and 7F). U87 cells likewise showed similar outcomes (Figure 7G and 7H). These results implied that the RANGAP1-SUMO1 axis played a key role in the regulation of Smad protein distribution in glioma cells, potentially affecting the TGF-β/Smad signaling pathway.

### Nuclear export of Smad4 is affected by RANGAP1-SUMO1

**Figure 8.**
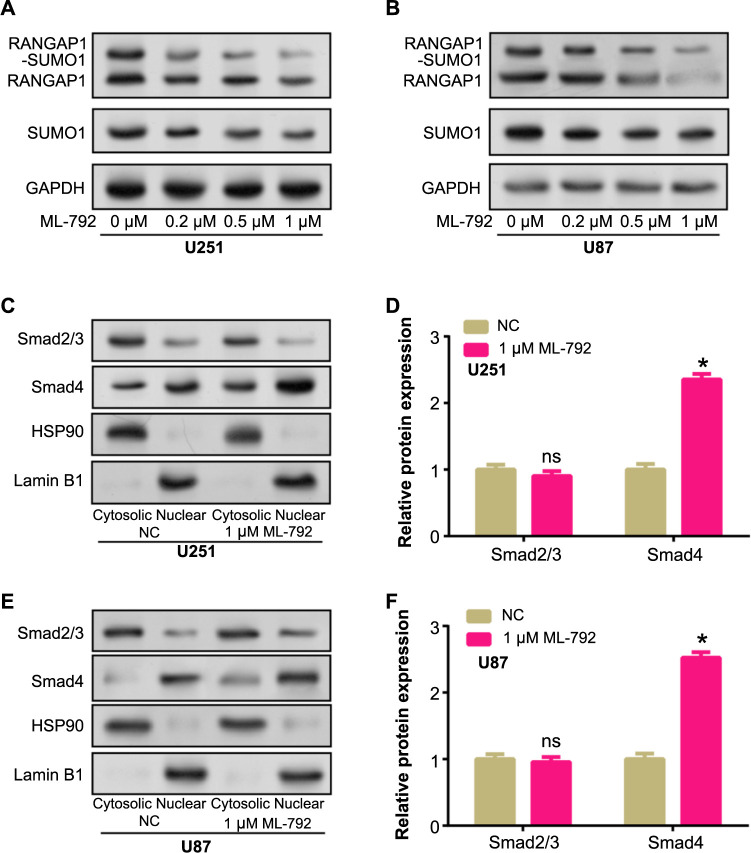
**RANGAP1-SUMO1 affects the nuclear export of Smad4.** (A and B) U87 and U251 cells were treated with a certain concentration gradient of ML-792 (inhibitor) for 2 h, and the inhibitory effect of ML-792 on RANGAP1-SUMO1 complex, RANGAP1 and free SUMO1 was evaluated by WB; (C–F) WB detection of nucleoplasmic expression levels of Smad2/3 and Smad4 after U87 and U251 cells were treated with 1 µM ML-792 for 2 h. HSP90 and Lamin B1 are reference proteins in the cytosol and nucleus, respectively. *P < 0.05. Each experiment was replicated three times. WB: Western blotting; Smad4: Sma and Mad-related protein 4; SUMO: Small Ubiquitin-like Modifier; ns: Not significant; NC: Normal control group.

To investigate the potential association between SUMOylation and RANGAP1 in the nuclear localization of SUMO1, the selective SUMOylation inhibitor ML-792 was employed. ML-792 specifically targets SUMO activation and has been previously demonstrated to inhibit the formation of RANGAP1-SUMO1 complexes. WB analysis showed a dose-dependent decrease in SUMOylated RANGAP1 levels with increasing concentrations of ML-792 in U251 and U87 cells but did not affect total RANGAP1 levels (Figure 8A and 8B). SUMO1 level also decreased, this verified the ability of ML-792 to block the SUMO process. To further investigate the effect of SUMO inhibition on Smad signaling, we evaluated the karyoplasmic distribution of Smad2/3 and Smad4 after ML-792 treatment. Following a 2-h course of therapy with 1 µM ML-792, accumulation of Smad4 was observed in the nuclei of both U87 and U251 cells, resulting in a significant increase in the proportion of Smad4 in the nucleus, while Smad2/3 showed no significant change (Figure 8C–8F). Selective nuclear translocation of Smad4 after SUMO inhibition suggests that SUMO might be involved in controlling the entry of Smad4 into the nucleus, thereby affecting TGF-β/Smad signaling pathway. These results suggest that inhibition of SUMO by ML-792 disrupts the normal nuclear output of Smad4 and may alter the functional dynamics of the TGF-β/Smad signaling pathway in glioma cells.

### RANGAP1 SUMOylation affects Smad4 to inhibit cell proliferation and migration

**Figure 9.**
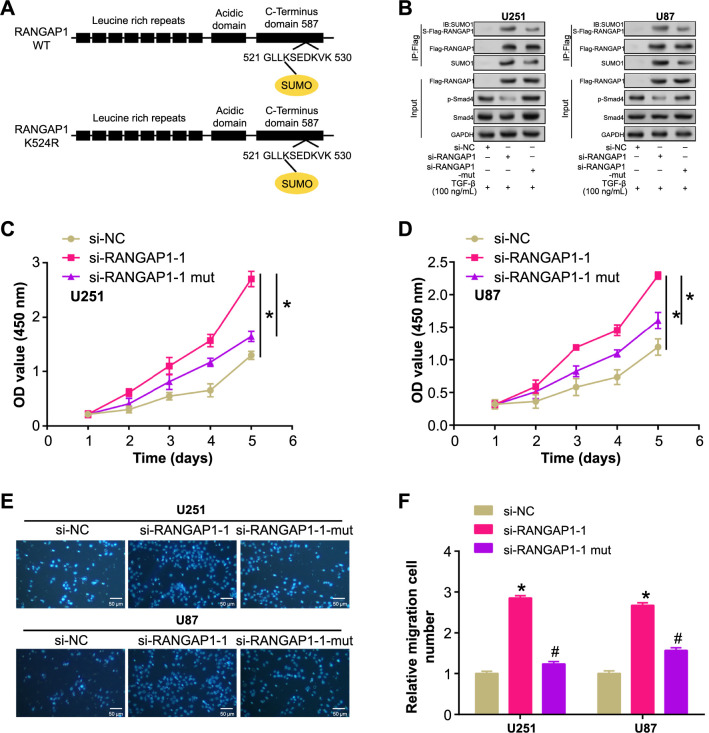
**RANGAP1 SUMOylation affects Smad4 to hinder cell proliferation and migration.** (A) Schematic representation of wild-type RANGAP1 (WT) and the RANGAP1 K524R mutant depicting the leucine-rich repeats, acidic domain, C-terminus, and SUMO modification site; (B) WB analysis after immunoprecipitation showing SUMO1 conjugation to RANGAP1 in U251 and U87 cells. Cells were transfected with si-NC, si-RANGAP1, or si-RANGAP1 in combination with SUMO1 under TGF-β stimulation. The phosphorylation status of Smad4 and total Smad4 levels were assessed; (C and D) Cell proliferation assays measuring the OD at 450 nm of U251 (C) and U87 (D) cells transfected with si-NC, si-RANGAP1-1, or si-RANGAP1-1-mut over five days; (E and F) Transwell migration assays of U251 and U87 cells transfected with si-NC, si-RANGAP1-1, or si-RANGAP1-1-mut. *P < 0.05, ^#^*P* < 0.05. Each experiment was replicated three times. WB: Western blotting; Smad4: Sma and Mad-related protein 4; TGF: Transforming growth factor; SUMO: Small Ubiquitin-like Modifier; OD: Optical density; NC: Normal control group.

The structural motifs of the wild-type and mutant RANGAP1 proteins are shown in Figure 9A. Eight leucine-rich repeat regions and high-acidic stretches are present in both proteins. In the mutant RANGAP1, lysine 524 is replaced by arginine, disrupting the SUMOylation site of RANGAP1. The K524R mutation impairs RANGAP1 SUMOylation levels, weakens the interaction between RANGAP1 and SUMO1, and enhances the phosphorylation of SMAD4 in U87 and U251 cells. After stimulation with TGF-β, increased Smad4 phosphorylation was observed in cells expressing wild-type RANGAP1. In contrast, the RANGAP1 K524R mutant exhibited reduced Smad4 phosphorylation, underscoring the importance of SUMOylation in TGF-β mediated Smad signaling (Figure 9B). CCK-8 assays revealed that si-RANGAP1-1 significantly promoted proliferation in U87 and U251 cells, while the K524 mutation weakened this effect (Figure 9C and 9D). Similarly, Transwell assays demonstrated that si-RANGAP1-1 significantly enhanced the migration ability of glioma cells, whereas the K524 mutation attenuated this enhancement (Figure 9E and 9F). These results emphasize the functional implications of the K524R mutation in RANGAP1, impacting cell proliferation and migration capacities in glioma cells.

## Discussion

Gliomas, including glioblastomas, represent a formidable challenge due to their infiltrative nature and limited treatment options [17]. Currently, the diagnosis of glioma mainly relies on neuroimaging technology, supplemented by histopathological analysis [18]. While these methods provide valuable insights into tumor localization and characteristics, they often lack specificity and sensitivity, leading to challenges in accurate diagnosis and monitoring of disease progression [19]. In recent years, targeted gene therapies have emerged as promising strategies for glioma treatment. By exploiting the molecular alterations specific to gliomas, such as mutations in the IDH gene and amplification of the EGFR gene, targeted therapies aim to selectively disrupt pathways crucial for tumor growth and survival [20]. Despite initial successes, challenges remain in achieving durable responses and overcoming resistance mechanisms associated with these therapies. Moreover, the five-year survival rate for patients with glioblastoma remains dismal, underscoring the urgent need for innovative approaches to improve prognosis and therapeutic outcomes [21]. In this context, the exploration of novel biomarkers holds significant promise for enhancing the precision of glioma diagnosis, guiding personalized treatment strategies, and predicting patient prognosis.

Building on our initial analysis of the GSE16011 dataset and TCGA-glioma dataset, we conducted differential gene screening and enrichment analysis on overlapping genes. Previous research by Patil et al. [22] established a link between glioma grade and the Fanconi Anemia pathway, specifically highlighting the re-expression of FANCD2. Inhibition of this pathway has emerged as a promising approach to improve the sensitivity of gliomas to chemotherapeutic agents. Additionally, a study by Yin et al. [23] shed light on the crucial role of miR-125a-3p in glioma development, directly regulating Nrg1 expression and impacting key processes like apoptosis, proliferation, and migration. This identifies miR-125a-3p as a potential diagnostic and therapeutic target for malignant glioma. Furthermore, the findings of Zou et al. [24] demonstrated that 2-Methoxyestradiol (2-ME) can enhance the effectiveness of radiotherapy in glioma cells by inducing G2/M cell cycle arrest, DNA damage, and activating ATM kinases. This substantiates its potential as a radiosensitizer in the treatment of glioblastoma multiform.

Our subsequent analysis focused on the prognosis and expression patterns of genes that overlap among glioma samples. Our findings revealed three genes that are prognostic indicators for glioma: *BRCA1, HDAC1,* and *RANGAP1*. Among them, some studies have shown that *BRCA1* may affect the development of glioma and patient prognosis by regulating the TGF-β/PI3K/AKT/mTOR signaling pathway [25]. Another study proposed that *BRCA1* expression is regulated by miR-212, affecting radiosensitivity and may affect the efficacy of radiotherapy for glioma [26]. *HDAC1* overexpression in gliomas is significantly associated with higher tumor grade, poorer prognosis, and increased immune infiltration, and is a key component of the prognostic signature. *HDAC1* can be a viable treatment target for gliomas as it has been demonstrated to influence cell invasion, proliferation, and apoptosis. Our analysis results showed that *BRCA1* and *HDAC1* were highly expressed in gliomas, while the expression results of *RANGAP1* were opposite. Since there are few studies on *RANGAP1* in glioma cell experiments, we selected *RANGAP1* as a hub gene for subsequent analysis.

The significance of *RANGAP1* in glioma is underscored by its multifaceted roles. Functioning as a GTPase-activating protein for Ran, RANGAP1 orchestrates crucial cellular processes, including nucleocytoplasmic transport, cell cycle progression, and mitotic spindle assembly [27]. By accelerating the GTP hydrolysis of Ran, *RANGAP1* contributes to proper protein localization and key cellular regulation. In addition to its canonical functions, *RANGAP1* is also involved in cell cycle control, microtubule organization, and mitosis, further highlighting its multifunctionality [28]. Examining its implications in glioma, *RANGAP1* emerges as a pivotal hub gene with potential prognostic significance and dynamic expression patterns. Studies by Zhu [29] demonstrate the intricate interplay of *RANGAP1* with SUMO1, mediated by Ubc9, in translocating from the cytoplasm to nuclear pore complexes. Concurrently, Zhao et al. [30] implicates *RANGAP1* in protein SUMOylation, offering a promising avenue for potential inhibitors. The regulatory role of *RANGAP1*, as highlighted by Zhang et al. [31] in intracranial aneurysm rupture, adds another layer of complexity, involving the myeloperoxidase (MPO)-modulating signaling pathway with miR-877-3p. Moreover, the study of Lin et al. [32] introduces a potential therapeutic strategy for glioblastoma through oridonin-induced downregulation of *RANGAP1*, leading to RNA accumulation and subsequent glioma cell apoptosis. Our study found that knocking down *RANGAP1* enhanced the proliferation, invasion, and migration abilities of U251 and U87 cells. Furthermore, the knockdown of *RANGAP1* disrupted cell cycle distribution and inhibited apoptosis in glioma cells. Collectively, understanding the multifaceted involvement of *RANGAP1* in glioma pathogenesis offers valuable insights for targeted interventions and enhances our comprehension of glioma progression and treatment strategies.

Expanding on the role of SUMO, in post-translational protein modification, it is a crucial participant in various cellular processes, covalently modifying target proteins and influencing their function, stability, or cellular localization [33]. The widespread impact of SUMOylation on biological activities, encompassing DNA repair, transcription, and maintenance of genome stability, underscores its significance in shaping cell physiology and disease pathways [34]. The research of Wong et al. [35] reveals a connection between SUMO-1 and specific lysosomes in neurodegenerative diseases marked by glial protein aggregation, exemplified in multiple system atrophy and progressive supranuclear palsy. This connection extends to glial cell models expressing α-synuclein, tau, or mutant huntingtin exon 1, suggesting a potential role for SUMO1 in lysosomal function in the context of glioma-associated protein aggregation. Additionally, Liu’s work sheds light on the impact of SENP1-mediated de-SUMOylation of SIRT1 in glioma development [36]. This process significantly influences cell activity, cycle progression, and apoptosis through the NF-κB pathway, proposing therapeutic implications for gliomas. Zhu et al.’s study revealed the interaction between Pin1 and ubiquitin specific peptidase 34 (USP34) in glioma stem cells. The research indicates that USP34 promoted the isomerization of Ubc9 and protein ubiquitination while removing the ubiquitination of Pin1, thus stabilizing Pin1. This interaction leads to global high SUMOylation of SUMO1-modified proteins, maintaining the tumorigenic potential of glioma stem cells [37]. In line with these findings, our investigation adds to the understanding of SUMO dynamics by revealing that *RANGAP1* undergoes SUMO1 conjugation modification, adding another level of complexity to the intricate landscape of glioma-associated protein modifications.

The Smad signaling pathway, a crucial intracellular cascade transmitting signals from TGF-β ligands, orchestrates diverse cellular processes, encompassing differentiation, growth, and apoptosis [38]. In the context of glioma, the investigation of Zhang et al. [39] discloses that diminished TGF-β receptor capability, induced by inhibitors, heightens TGF-β ligand synthesis in stem/progenitor cells, fostering proliferation through non-Smad pathways like mTOR and NF-κB. At the same time, the research of Yao et al. [40] highlights the role of SECTM1 in glioma progression and activates the TGFβ1/Smad signaling pathway, making it a prospective therapeutic target and biomarker. In addition, the findings of Zhang et al. [41] elucidated that the Smad pathway is involved in TGF-β2-induced autophagy, which is critical for glioma invasion by affecting processes such as epithelial–mesenchymal transition and mitochondrial transport, as well as maintaining autocrine loops. Building on these findings, our research demonstrates that *RANGAP1* glycosylation inhibits Smad4, thereby obstructing cell proliferation and migration. We also discovered that reduced levels of SUMO1 impact the distribution of Smad4 between the nucleus and cytoplasm. Moreover, the RANGAP1-SUMO1 complex influences the nuclear export of Smad4. These insights highlight the complex interplay between SUMOylation, the Smad signaling pathway, and glioma progression, providing a more comprehensive understanding of potential therapeutic targets for glioma treatment.

## Conclusion

Overall, this study demonstrates a comprehensive understanding of the complex relationship between SUMOylation, the Smad signaling pathway, and glioma progression. The discovery of key prognostic genes, particularly *BRCA1*, *HDAC1*, and *RANGAP1*, highlights their significant impact on glioma survival. Notably, RANGAP1 emerged as a key regulator affecting crucial cellular processes in glioma cells, shedding light on its potential therapeutic relevance. SUMO1 conjugation serves as a major modulator of *RANGAP1*, affecting the distribution of Smad4 across the nucleus and cytoplasm and impacting the TGF-β/Smad signaling pathway. The use of SUMOylation inhibitors such as ML-792 has shown their potential therapeutic utility in regulating this system. The K524R mutation in *RANGAP1* further highlighted its crucial function in cell proliferation and migration, providing insights into glioma biology and opening up new options for targeted therapies.

## Supplemental data

Supplementary table is available at the following link: https://www.bjbms.org/ojs/index.php/bjbms/article/view/10443/3289

## Data Availability

The data that support the findings of this study are available on request from the corresponding author upon reasonable request.
